# A Condition-Aware Shading Domain-Adaptive Framework for Robust Chlorophyll Inversion Across Shade Managements in *Hopea hainanensis*

**DOI:** 10.3390/plants15081236

**Published:** 2026-04-17

**Authors:** Lin Chen, Xiaoli Yang, Xiaona Dong, Ling Lin, Mengmeng Shi, Feifei Chen, Chuanteng Huang, Huilin Yu, Ying Yuan, Miaoyi Han

**Affiliations:** 1 Hainan Academy of Forestry (Hainan Academy of Mangrove), Haikou 571100, China; 2 The Innovation Platform for Academicians of Hainan Province, Haikou 571100, China; 3 Research Institute of Forest Resource Information Techniques, Chinese Academy of Forestry, Beijing 100091, China; 4 Precision Agriculture Lab, School of Life Sciences, Technical University of Munich, 85354 Freising, Germany

**Keywords:** *Hopea hainanensis*, domain adaptation, transfer learning, conditional shift, deep learning, shade management, chlorophyll content

## Abstract

Shade management, which is widely adopted in cultivation and understory regeneration, alters plant light environments, thereby degrading the trait inversion performance and posing a key challenge in plant phenotyping. To address this issue, this study reframed chlorophyll retrieval of *Hopea hainanensis* under shade management as an illumination-regime-dependent conditional domain shift problem, and developed a condition-aware domain adaptation framework (CAI-DAI) tailored to this setting. The results showed that chlorophyll content increased with shading intensity, accompanied by clear differences in canopy spectral distributions among shading levels, supporting the presence of condition-dependent variation under shade management. Model comparisons showed that CA-IE and CAI-DAI, which integrate conditional encoding and conditional alignment, performed better than the comparative models across fine-tuning ratios from 30% to 70%. Among them, CAI-DAI achieved the best and most stable performance, with test MAE ranging from 4.355 to 4.774 μg·cm^−2^ and nRMSE ranging from 16.4% to 18.2%, and R^2^ ranging from 0.456 to 0.585. Further evaluation at individual shading levels (S1–S4) showed that CAI-DAI produced narrower error ranges than CA-IE. It also showed smaller error fluctuations under most fine-tuning ratios. These results demonstrate that the proposed framework effectively improves robustness under heterogeneous shading conditions and limited labeled samples, providing methodological support for chlorophyll monitoring and decision-making related to shade management.

## 1. Introduction

Hyperspectral remote sensing has emerged as a core sensing technology for high-throughput phenotyping of plant physiological and biochemical functions, offering substantial application potential in agricultural management, forest cultivation, and ecological assessment [1,2,3]. Particularly in forestry cultivation practices, shading regulation, as a key artificial management measure, is widely used to modulate the microenvironment of seedlings, thereby improving survival rates and enhancing their adaptability to future understory regeneration conditions [4,5]. However, differences in light intensity and spectral composition induced by varying shading levels can alter the radiative transfer process of canopy reflectance signals [6,7]. Since hyperspectral signals are inherently modulated by incident radiation conditions, the generalization performance of physiological trait inversion models constructed based on hyperspectral data in current studies is usually highly dependent on the consistency of observation conditions. When substantial illumination mismatches exist between the training data and the actual application scenarios (e.g., different shading levels), even if the model performs excellently under a single observation condition, its cross-condition transferability may decline substantially [8,9]. Therefore, how to improve the robustness and transferability of inversion models under the highly heterogeneous light background caused by artificial shading constitutes a critical challenge for promoting the transformation of hyperspectral physiological trait estimation technology from regional controlled experiments to practical forestry applications.

*Hopea hainanensis*, as a key species for tropical rainforest restoration and the artificial cultivation of rare tree species [10,11,12], generally relies on shade management at the seedling stage to regulate the light environment, avoid photoinhibition, and promote its physiological adaptation [13,14]. In this context, chlorophyll content, as a core physiological indicator reflecting plant photosynthetic capacity and nitrogen nutrition status, exhibits dual sensitivity to light conditions [2,15]. Physiologically, plants regulate chlorophyll content to optimize photosynthetic processes and thereby acclimate to contrasting light environments [16,17,18]. Spectrally, the characteristic absorption features of chlorophyll are highly susceptible to interference induced by changes in light conditions [2,19]. Therefore, achieving stable and accurate inversion of chlorophyll content under shade management conditions not only facilitates the understanding of the light adaptation mechanism of *H. hainanensis* but also provides technical support for optimizing cultivation and management strategies for this high-value species.

From the modeling perspective, radiative transfer models (RTMs, e.g., PROSAIL) provide a solid physical model foundation for the inversion of chlorophyll content using hyperspectral data by simulating the physical relationships between leaf biochemical parameters and spectral reflectance [6,20,21]. However, standalone RTM inversion approaches, such as LUT-based spectral matching, can be sensitive to prior constraints and inversion settings, which limit their stability in practical applications. Hybrid strategies that couple RTMs with machine learning methods have been widely adopted in recent years to retrieve plant traits such as chlorophyll, nitrogen, leaf area index, and biomass [22,23,24,25]. By integrating physical prior constraints with data-driven modeling, these approaches have mitigated overfitting issues under small-sample conditions to some extent and enhanced model transferability across different environmental backgrounds. However, RTMs are typically formulated under assumptions of uniform canopy structure and relatively stable light conditions [21], whereas light in operational forestry settings with shade management is far from uniform and often exhibits pronounced spatial heterogeneity. Observed canopy reflectance generally comprise a mixture of contributions from directly illuminated and shaded leaves [26,27]. The combined effects of factors such as changes in the direct–scattered ratio, reconstruction of multiple scattering paths, and differences in signal-to-noise ratio (SNR) can yield distinct spectral reflectance patterns even when leaf biochemical properties remain consistent [28,29,30]. This distribution shift induced by changes in light regimes essentially constitutes a light condition-dependent domain shift, which may lead to systematic biases when retrieval models developed under uniform illumination assumptions are applied across different shading conditions.

To mitigate the effects of illumination variability on spectral reflectance, a range of strategies have been proposed [31,32,33]. Some studies have employed spectral normalization or shadow correction techniques to reduce the interference of brightness differences in model predictions [33,34,35]. More recently, with the development of deep learning and domain adaptation, increasing attention has been given to feature-level distribution alignment to improve model transferability across heterogeneous data sources [36,37]. For example, Anand et al. [37] improved the transferability of a deep learning model across agricultural observation scenarios through domain adaptation and fine-tuning, whereas Weng et al. [38] addressed cross-domain inconsistency in hyperspectral–multispectral fusion by introducing dual-domain alignment in temporal, spatial, and spectral spaces. In addition, Wang et al. [39] showed that joint correlation alignment can enhance adaptation performance in multitemporal hyperspectral remote sensing, highlighting the importance of temporal domain discrepancy. Nevertheless, these approaches still show notable limitations under shade management conditions. Most existing domain adaptation studies define domains according to factors such as acquisition scenario, sensor or modality, and observation time, while rarely treating shading-induced light-condition differences themselves as the core organizing variable of domain structure. Consequently, in the context of shade management, the conditional domain structure induced by differences in light regimes has not yet been systematically modeled or interrogated, representing a potentially important gap in current hyperspectral trait retrieval research.

To address the aforementioned challenges, in this study, chlorophyll retrieval under shade management was formulated as a condition-dependent domain adaptation problem, in which discrete shading regimes induce structured shifts in both spectral distributions and the spectrum–chlorophyll relationship. Based on this view, we propose a Condition-Aware Illumination Domain-Adaptive Inversion (CAI-DAI) framework to retrieve chlorophyll content in *H. hainanensis* under different shade management, and conduct ablation analyses to validate the effectiveness of this framework in improving the generalization stability across light conditions. Specifically, different shading levels are defined as conditional domains with distinct light regime characteristics. A condition-aware mechanism is incorporated into the inversion process to capture the influence of shading levels on the spectral–chlorophyll relationship, while a domain feature alignment strategy is employed to mitigate the distribution differences between different shading levels. This approach offers a novel perspective for enhancing the robustness of hyperspectral chlorophyll inversion in heterogeneous light environments.

## 2. Results

### 2.1. Effects of Shade Management on Leaf Chlorophyll Content in H. hainanensis

**Figure 1:**
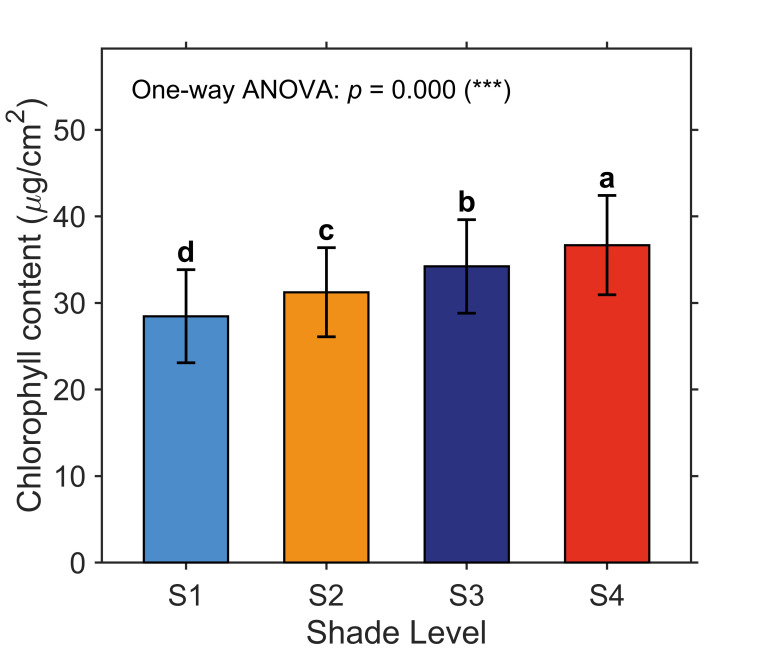
Changes in leaf chlorophyll content of *H. hainanensis* under different shading levels. Error bars indicate standard deviation. Different lowercase letters (a–d) above the bars indicate significant differences among shading levels according to Tukey’s HSD test. The one-way ANOVA was significant (***, *p* < 0.001).

Figure 1 shows that the leaf chlorophyll content of *H. hainanensis* increased with increasing shading level, and a one-way ANOVA confirmed that the differences were statistically significant. The mean chlorophyll contents at shading levels S1–S4 were 28.463, 31.238, 34.226, and 36.681, respectively. Furthermore, Tukey’s HSD post hoc test indicated that the chlorophyll content differed significantly between every pair of shading levels.

### 2.2. Differences in Spectral Responses Across Shading Levels

**Figure 2:**
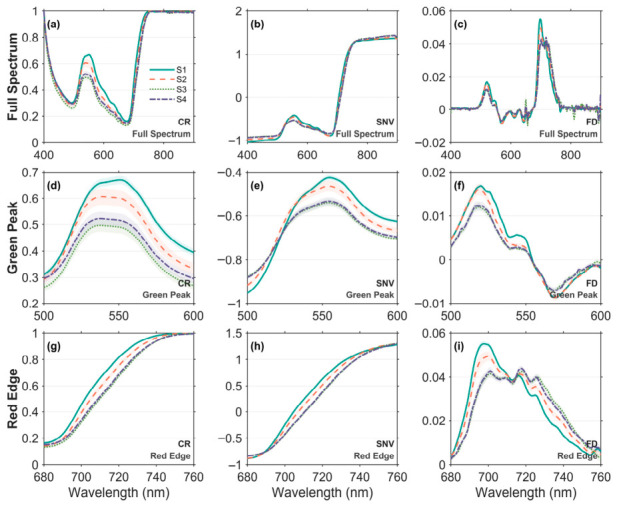
Mean spectra of the full, green peak, and red edge regions under different shading levels after CR, SNV, and FD transformations. (**a**) full spectrum after CR; (**b**) full spectrum after SNV; (**c**) full spectrum after FD; (**d**) green peak region after CR; (**e**) green peak region after SNV; (**f**) green peak region after FD; (**g**) red edge region after CR; (**h**) red edge region after SNV; (**i**) red edge region after FD.

Figure 2 shows the response of the average spectral characteristics of the *H. hainanensis* canopy under different shading levels after CR, SNV, and FD transformations. To further examine the responses of key spectral regions, in addition to the full-waveband spectra (400–900 nm; Figure 2a–c), the green peak region (500–600 nm; Figure 2d–f) and the red-edge region (680–760 nm; Figure 2g–i) were enlarged separately.

Across the full spectral range, differences among shading treatments were most pronounced in the green peak and red-edge regions. In the green peak region, spectral reflectance decreased with increasing shading after CR, SNV, and FD transformations. Notably, the CR spectrum still maintained good discrimination between moderate shading (S3) and heavy shading (S4), whereas the SNV and FD spectra overlapped considerably between S3 and S4. In the red-edge region, CR and SNV spectra also decreased overall with increasing shading. For the FD spectrum, its variation trend showed opposite characteristics before and after the red-edge inflection point (REIP, approximately 715–720 nm): the spectral value decreased with increasing shading before the inflection point, but increased after the inflection point. Consistent with the green peak region, the spectral characteristics of S3 and S4 were similar under all transformations.

**Figure 3:**
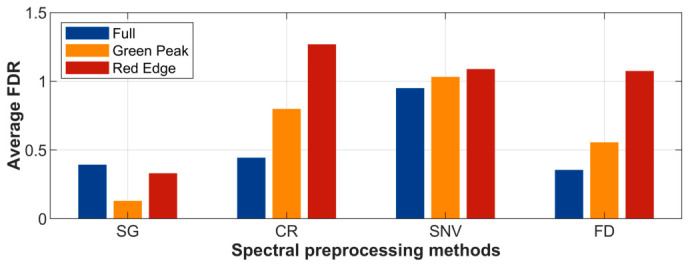
FDR values for each spectral region under different spectral transformations.

The Fisher Discriminant Ratio (FDR) was calculated for the three preprocessed spectra according to shading levels, and compared with the FDR of the original reflectance after SG smoothing (Figure 3). Results showed that the three preprocessing methods improved the separability of spectral variations among different shading levels in the green peak and red-edge regions, thus enhancing the spectral response to shading changes.

To further analyze differences in spectral distribution characteristics of key sensitive bands under different shading levels, permutational multivariate analysis of variance (PERMANOVA) was conducted on the three transformed spectra (CR, SNV, and FD) for the green peak and red edge regions. The number of permutations was set to 999. The analysis produced the pseudo-F value, which reflects the magnitude of between-group differences relative to within-group dispersion, the significance level based on permutations (*p*_perm_), and the effect size (R^2^_perm_) (Table 1).

Results showed that the spectral distribution differences among shading levels in both the green peak and red-edge regions reached the minimum distinguishable *p*-value (*p*_perm_ = 0.001) under the set permutations, indicating significant differences. This suggests that the overall spectral shape differed significantly under shading treatments, rather than only random differences at individual bands. Further comparison revealed that pseudo-F and R^2^_perm_ values were higher in the red edge region than in the green peak region for all three spectral transformation methods, indicating a stronger response of the red edge region to shading gradients. Specifically, the effect sizes of CR, SNV, and FD in the red edge region were 0.398, 0.423, and 0.349, respectively, which were higher than the corresponding values of 0.311, 0.303, and 0.227 in the green peak region.

Among them, SNV achieved the highest effect size (R^2^_perm_ = 0.423) and the highest pseudo-F value (76.013) in the red-edge region, demonstrating the best performance in enhancing spectral responses under different shading levels.

### 2.3. Overall Performance Comparison of Chlorophyll Retrieval Models

Test accuracies (nRMSE and MAE) of the five model configurations under different fine-tuning set ratios (30–70%) are listed in Table 2, based on spectral features selected via a two-stage screening procedure. The retained features showed clear spectral-region specificity across preprocessing methods. Features selected from CR were mainly concentrated in the red-edge region, whereas FD retained features primarily from the green peak reflectance region and the red-edge region, both of which are closely related to chlorophyll estimation. In addition, SNV contributed complementary information from the blue absorption region and the near-infrared region. The detailed feature-selection results are provided in the Figure S1.

**Figure 4:**
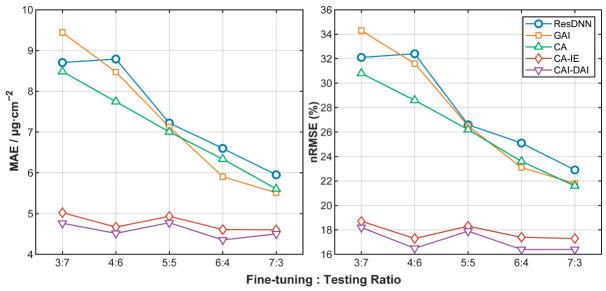
Changes in test accuracy (nRMSE and MAE) of the five models (ResDNN, GAI, CA, CA-IE, CAI-DAI) across different fine-tuning set ratios (30–70%).

The trends of test accuracy with fine-tuning set ratio are illustrated in Figure 4. Moreover, the baseline ResDNN model showed clear sensitivity to the amount of target-domain fine-tuning data. The MAE decreased from 8.706 at the 3:7 split to 5.951 at the 7:3 split, whereas the corresponding change for CAI-DAI was much smaller, from 4.761 to 4.502. A similar trend was also observed for nRMSE, further indicating that the condition-aware models were less dependent on fine-tuning sample size than the baseline models. This ratio-dependent contrast suggests that the performance of the baseline models was more strongly constrained by target-domain annotation availability, whereas CA-IE and CAI-DAI were able to maintain comparatively stable performance under more limited fine-tuning data. In terms of overall accuracy, the CA-IE and CAI-DAI models, which integrate conditional domain adaptation and conditional encoding, achieved higher accuracy than the ResDNN, GAI, and CA models. Among them, the CAI-DAI model performed best and maintained high and stable accuracy across all fine-tuning set ratios. Its MAE ranged from 4.355 to 4.774 μg·cm^−2^, representing a reduction of 28.4–49.1% compared with the baseline ResDNN model. Its nRMSE remained between 16.4% and 18.2%, corresponding to a decrease of 24.3–48.2% relative to ResDNN. The CA-IE model ranked second, with MAE values ranging from 4.600 to 5.024 μg·cm^−2^ and nRMSE values ranging from 17.3% to 18.7%, which were slightly higher than those of the CAI-DAI model. At the same time, Table 2 also shows that several comparative models still produced relatively high errors under some split settings, especially when the fine-tuning ratio was low, indicating that robust chlorophyll retrieval across shading conditions remained challenging under limited target-domain supervision. At the same time, Table 2 shows that some comparative models still had relatively high errors under lower fine-tuning ratios. For example, at the 3:7 split, the MAE values of ResDNN, GAI, and CA were 8.706, 9.442, and 8.485 μg·cm^−2^, respectively, with corresponding nRMSE values of 32.1%, 34.3%, and 30.8%, all clearly higher than those of CA-IE and CAI-DAI.

In terms of accuracy variation, the ResDNN, GAI, and CA models showed a strong dependence on fine-tuning set size. As the fine-tuning ratio increased, the test performance of these three models improved continuously. In contrast, the test accuracies of the CA-IE and CAI-DAI models varied only slightly with the fine-tuning set ratio, indicating more stable error levels.

**Figure 5:**
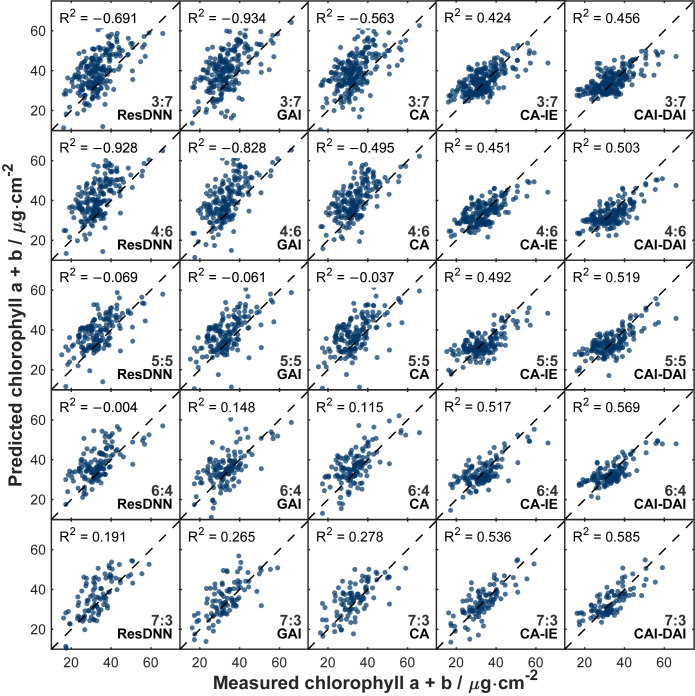
Scatter distributions of predicted and measured chlorophyll content for the five models (ResDNN, GAI, CA, CA-IE, CAI-DAI) different fine-tuning set ratios (30–70%).

Figure 5 shows the scatter distributions of predicted versus measured chlorophyll contents under different model configurations and fine-tuning set ratios. R^2^ values are reported in this study; under some low-ratio settings, negative R^2^ values occurred when model predictions were poorer than a simple mean-value baseline on the same test set [40]. Within the fine-tuning ratio range of 30–70%, the ResDNN, GAI, and CA models exhibited clear systematic overestimation in chlorophyll prediction, with most scatter points distributed above the 1:1 line. When the fine-tuning ratio increased to 60–70%, the degree of overestimation was reduced, although systematic bias remained. In contrast, the CA-IE and CAI-DAI models showed no obvious systematic overestimation or underestimation across all fine-tuning set ratios, and their scatter points were distributed more closely around the 1:1 line. Nevertheless, some residual dispersion around the 1:1 line was still present, indicating that prediction error was reduced but not completely eliminated. At a fine-tuning ratio of 70%, the CAI-DAI model achieved the best predictive performance, with an R^2^ value of 0.585 and the lowest scatter dispersion.

### 2.4. Model Generalization Across Shading Levels

**Figure 6:**
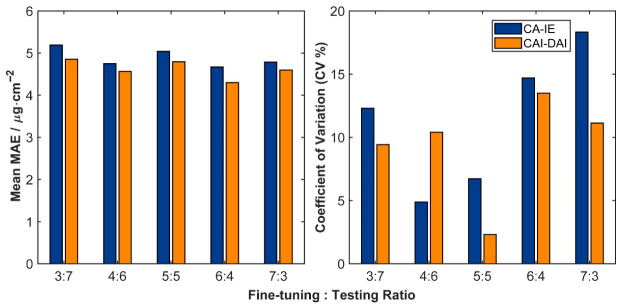
Mean MAE and coefficient of variation across shading levels for the CA-IE and CAI-DAI models under different fine-tuning set ratios.

Besides overall accuracy, the generalization performance of the models under different shading levels was also a key indicator for evaluating their applicability. The above results showed that the CA-IE and CAI-DAI models achieved higher overall accuracy than the other comparative models. Therefore, the subsequent analysis focused on the performance of these two models under different shading conditions. The MAE values for chlorophyll content estimation in *H. hainanensis* under each shading level (S1–S4) were calculated for the CA-IE and CAI-DAI models at different fine-tuning set ratios. The detailed results are presented in Table 3. On this basis, the mean MAE values and coefficients of variation under each shading level were further computed to evaluate the estimation stability of the models within a single shading condition (Figure 6).

The results showed that the MAE ranges of the CA-IE model at the four shading levels were 4.072–4.581 (S1), 3.979–6.058 (S2), 4.622–5.578 (S3), and 4.396–5.803 (S4) μg·cm^−2^. Accordingly, the MAE ranges of the CAI-DAI model were 4.129–4.704 (S1), 3.504–5.182 (S2), 4.275–4.931 (S3), and 4.835–5.307 (S4) μg·cm^−2^. In terms of mean MAE across shading levels, CAI-DAI showed lower values than CA-IE under all fine-tuning set ratios. In terms of stability, the coefficients of variation in MAE summarized by shading level showed that the CV values of CAI-DAI were lower than those of CA-IE under most fine-tuning set ratios. This finding indicates that CAI-DAI produced smaller error fluctuations and therefore achieved more stable performance under different shading conditions.

## 3. Discussion

### 3.1. Differences in Chlorophyll and Canopy Spectra Induced by Shade Management

This study reveals significant differences in both spectral responses and chlorophyll content of *H. hainanensis* seedlings under varying shading levels. At the physiological level, chlorophyll content increased significantly with increasing shading (Figure 1, Table 1). This result is consistent with the light-adaptive strategy of shade-tolerant tree species: under low-light conditions, plants increase chlorophyll content to improve light-harvesting efficiency and maintain photosynthetic capacity [16,17,18]. Our findings are consistent with those of Xie et al. [41] in seedlings of two subtropical shade-tolerant tree species. They reported sustained and significant increases in leaf chlorophyll a and b contents with decreasing light intensity. This study further validates the applicability of this pattern in *H. hainanensis*, demonstrating that *H. hainanensis* seedlings are highly sensitive to light gradients in their physiological responses.

At the spectral response level, systematic differences exist in spectral characteristics among shading levels, and these differences can be further enhanced by spectral transformations. After CR, SNV, and FD transformations, spectral reflectance in the green peak and red-edge regions showed regular changes with increasing shading (Figure 2). These results are consistent with previous studies that spectral transformations enhance spectral absorption and reflection features, thereby improving sensitivity to vegetation biogeochemical parameters [33,42,43]. More importantly, PERMANOVA confirmed highly significant differences in spectral responses among shading levels in both the green peak and red-edge regions (*p*_perm_ = 0.001). This indicates that the spectral changes induced by shade management are not random noise or simple brightness disturbances, but systematic band-dependent shifts. These patterns suggest that shading effects in this study were not simply expressed as uniform brightness changes, but were associated with structured shifts in physiologically relevant spectral regions.

Based on the above, shade management leads to an illumination-regime-dependent domain shift with structural characteristics. Different light environments induce differentiation in plant physiological status, which further results in systematic shifts in spectral responses. This also implies that different shading conditions represent distinct statistical subdomains, rather than random fluctuations within the same distribution [7]. This definition provides an empirical foundation for the subsequent introduction of domain adaptation and conditional modeling.

### 3.2. Light-Dependent Mapping and Conditional Shift Under Shade Management

After confirming the distinct differentiation of spectral distributions caused by shading levels, a more direct question arose: whether these spectral differences further manifested as cross-condition instability in the spectrum–chlorophyll mapping relationship. The model comparison and error patterns observed in this study provided suggestive support for this possibility. In the model comparison analysis, the baseline ResDNN model, which did not account for shading conditions, showed limited generalization ability across shading scenarios. Even with the introduction of global statistical alignment methods, the GAI model showed only limited performance improvement (Figure 4). By contrast, models explicitly incorporating conditional information and conditional alignment (CA-IE and CAI-DAI) consistently outperformed the other model configurations under different fine-tuning sample ratios (Table 2; Figure 4 and Figure 5). These findings are consistent with the presence of condition-dependent changes in the spectrum–chlorophyll relationship under different shading environments, rather than supporting a purely global distribution shift alone [44,45].

From the perspective of plant spectral mechanisms, this interpretation is reasonable. Shading not only changes the intensity of incident irradiance, but also alters the direct-to-diffuse ratio, shadow background contribution, and multiple scattering paths within the canopy [6,7]. These effects further influence the expression of absorption features at the canopy scale. At the same time, increases in chlorophyll content often occur together with changes in other physiological and structural traits [17,46]. This leads to variation in the sensitivity of red-edge and near-infrared scattering features across light environments [2,19]. Together, these factors may contribute to different spectral response sensitivities to chlorophyll content under different light environments, that is, conditional changes in the spectrum–chlorophyll mapping.

In summary, this study suggests that shade management does not merely introduce spectral noise. Instead, it is associated with changes in plant physiological status and with corresponding shifts in the spectrum–chlorophyll relationship. Cross-shading inversion may therefore be better understood as involving both global and condition-dependent domain discrepancies. The CA-IE and CAI-DAI models leverage the advantages of conditional encoding and conditional alignment. They learn cross-shading transferable representations and perform conditional correction of representation distributions and predictions under different shading regimes. This reduces systematic bias caused by differences in light regimes. As reflected in the scatter patterns and error metrics, this helped reduce systematic bias to some extent, although some residual bias and dispersion still remained under certain settings.

### 3.3. Heterogeneity and Uncertainty in Shading Responses

Although the CA-IE and CAI-DAI models, which integrate domain adaptation and conditional encoding, showed better overall performance than the other models (Table 2; Figure 4), further analysis by shading level revealed that the errors were not evenly distributed across light conditions (Table 3). For instance, the CA-IE model showed a wide range of MAE fluctuations under moderate shading (S2) and heavy shading (S4), with values of 3.979–6.058 for S2 and 4.396–5.803 for S4 (μg·cm^−2^). This suggests that its estimation stability still faces challenges under certain conditions. This phenomenon can be interpreted from the perspective of heterogeneous light responses among individual seedlings. In a real nursery environment, even within the same shading level, seedlings may differ in leaf age, health status, micronutrient conditions, and local microenvironment [13,27,47]. Such variation in physiological status at the individual level increases uncertainty in the spectrum–Cab relationship. In addition, some uncertainty may also arise from canopy-scale heterogeneity within individual seedlings, local differences in light interception and shadow background, and the imperfect correspondence between canopy spectral observations and chlorophyll reference measurements. Physiological responses may become more complex under moderate shading, where light adaptation and light stress can coexist [47,48].

The advantage of the CAI-DAI model over CA-IE lies in its introduction of an uncertainty-weighting mechanism. As shown in Figure 6, under most fine-tuning ratios, the coefficient of variation of mean absolute error across shading levels is lower for CAI-DAI than for CA-IE. This indicates smaller error fluctuations and more stable estimation. These results suggest that the uncertainty-weighting mechanism is not merely a statistical optimization technique [49]. Rather, it is consistent with a more robust treatment of condition-dependent heterogeneity during training. It effectively captures heterogeneous light responses at the seedling stage. It also reduces model overfitting to extreme or abnormal individuals, thereby improving overall model robustness under complex shading conditions.

### 3.4. Robustness Under Limited Sample Conditions

The practical feasibility of models relies heavily on their ability to adapt to limited labeled samples [50]. In this study, the sensitivity of each model to small-sample conditions was systematically evaluated by setting different fine-tuning ratios (30–70%). Figure 4 and Table 2 clearly show a key trend: The performance of ResDNN, GAI, and CA models depends strongly on fine-tuning sample size. Accuracy drops as training samples decrease. This is consistent with previous studies on trait inversion models incorporating RTM-simulated data, which reported similar trends in model accuracy across different dataset sizes [23,51]. In contrast, the test accuracy of CA-IE and CAI-DAI models changes much more gently with fine-tuning ratio. Even with only 30% fine-tuning data, their nRMSE and MAE remain at low levels. The scatter distribution in Figure 5 supports this observation. Even at a 30% fine-tuning ratio, predictions from the CAI-DAI model lie close to the 1:1 line, with no systematic overestimation. Within the scope of the present dataset, these results suggest that the condition-aware models were less sensitive to limited target-domain labels than the baseline models. They also provide useful methodological insight for controlled forestry and nursery monitoring scenarios where labeled measurements are difficult to acquire.

In practical stand monitoring or nursery management, obtaining large numbers of high-quality measurements (spectrum and chlorophyll content) is often costly and time-consuming [52,53,54]. This study demonstrates that advanced conditional domain adaptation models such as CAI-DAI can achieve stable and accurate chlorophyll inversion under complex shading conditions with effective calibration using limited samples. This provides a promising technical route for large-scale, low-cost seedling monitoring, precision fertilization, and seedling quality regulation via remote sensing. However, the interpretation of such robustness should remain within the scope of the present controlled dataset, and further validation across broader datasets and operational environments is still needed.

### 3.5. Limitations and Future Perspectives

This study verifies the advantages of the conditional domain adaptation model (CAI-DAI) for chlorophyll inversion across shading scenarios. Meanwhile, several limitations exist and require further investigation in future work. Recent review studies have also highlighted the expanding role of deep learning in plant phenotyping, while also pointing to the importance of scenario-specific model adaptation [55]. It should also be noted that the performance gains achieved by CAI-DAI come with increased computational cost, because the framework integrates multiple modules, including condition encoding, feature adaptation, and uncertainty-aware learning.

To facilitate controlled experimental conditions and mechanism analysis, this study divides shading treatments into four discrete levels (S1–S4) for modeling. However, in real agricultural and forestry systems, especially in agrivoltaics or heterogeneous agroforestry settings, shading often shows clear temporal dynamics and continuous light gradients rather than fixed discrete levels. Therefore, future studies can introduce continuous light indicators as conditional variables for the model. These indicators may include daily total photosynthetically active radiation, red/far-red ratio, or the ratio of direct to diffuse light. Such an approach can promote the model from discrete light-level modeling to adaptive modeling for continuous light environments. It can also better characterize the physiological responses of plants to light gradients and improve the ecological realism and field applicability of the model.

Moreover, this study focuses on seedlings of *Hopea hainanensis*, a typical shade-tolerant tree species. Tree species with different photo-ecological types (e.g., heliophytes or mesophytes) show different regulatory strategies in response to light stress [47]. Thus, future research can systematically test the modeling framework proposed in this study across multiple photo-ecological types, including heliophytes, mesophytes, and shade-tolerant species. This will help evaluate and improve the generalization ability of the model in different forest types. It will also enhance its adaptability to complex forestry applications from a methodological perspective. Accordingly, it can provide stronger theoretical support for universal intelligent monitoring toward precision forestry.

## 4. Materials and Methods

### 4.1. Experimental Design

**Figure 7:**
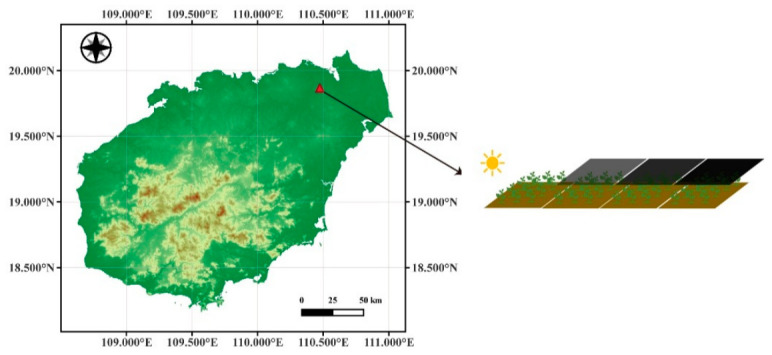
Experimental site and design. The map colors represent elevation variation, and the red triangle indicates the location of the experimental site.

A controlled shading experiment was established at an experimental site in Yunlong Town, Haikou City, China (Figure 7). Shading gradients were created by suspending black polyethylene shade nets (Lvandi, Greenland Shade Co., Taizhou, China) approximately 2 m above ground. The experiment employed a completely randomized block design comprising four shading levels, specified by nominal shade intensity (and corresponding nominal light transmittance): S1 (full-sun control; 0% shade, 100% transmittance), S2 (light shade; 30% shade, 70% transmittance), S3 (moderate shade; 60% shade, 40% transmittance), and S4 (heavy shade; 90% shade, 10% transmittance). To quantify actual illumination conditions, radiance from a calibrated white reference panel was recorded with a hyperspectral sensor and used to estimate incident irradiance and derive photosynthetically active radiation (PAR). These measurements were then used to calibrate the effective transmittance of each treatment, yielding approximate values of 100%, 60%, 30%, and 10% for S1–S4, respectively. All experimental plots were managed consistently throughout the experiment, including routine weeding and irrigation.

### 4.2. Field Data Acquisition

Ground-based canopy hyperspectral measurements were acquired in two key sampling campaigns, conducted in November 2021 and June 2023. The measurements were performed using a portable field spectroradiometer (PSR 1100f, Spectral Evolution, Lawrence, MA, USA), covering a spectral range of 325–1100 nm with a 3 nm spectral resolution and 1 nm sampling interval. The optical fiber was positioned vertically downwards, with a 25° field of view. For each selected plant, ten rapid successive scans were collected and averaged to produce the raw reflectance spectrum. White reference panel readings were taken in parallel to enable radiometric calibration. Considering the requirement to balance the representativeness of healthy plants and the limitations of practical work intensity, it was not feasible to conduct measurements on every sapling that had been planted. Instead, from each experimental plot, individual saplings showing robust growth and no obvious pest or disease symptoms were chosen randomly as observation samples. Across the two measurement campaigns, valid canopy hyperspectral data were collected from a total of 315 separate individual plants.

Subsequent to the hyperspectral measurements, the leaf chlorophyll content of all 315 tropical tree samples was quantified with a portable chlorophyll meter, and each measurement was directly linked to the corresponding canopy hyperspectral data. As SPAD values only reflect relative chlorophyll content, it is usually necessary to convert them into absolute chlorophyll density (chlorophyll a and b; μg·cm^−2^) through empirical conversion methods. Previous studies have confirmed that there is a strong non-linear correlation between SPAD values and chlorophyll content. In this study, given that the research objects are tropical tree species, we applied the calibration function proposed by Coste et al. [56], derived from measurements across 13 tropical tree species (Equation (1)). This relationship has been suggested to be broadly transferable among tropical taxa and has also demonstrated good performance in chlorophyll-related applications for other plant species [57,58]. Accordingly, the chlorophyll content values reported in the following analyses are calibrated values.

(1) $$CHL=\frac{117.1\times SPAD}{148.84-SPAD},\;\;R^{2}=0.89$$

### 4.3. Spectral Preprocessing and Feature Selection

**Figure 8:**
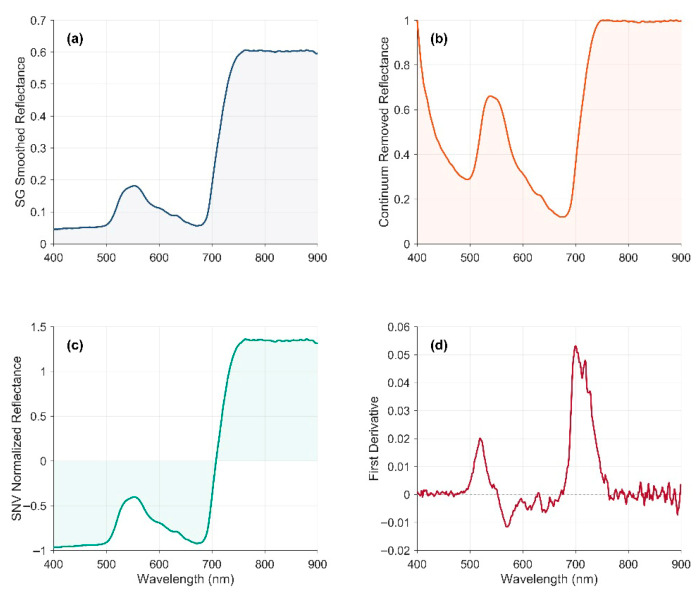
Representative spectra after SG smoothing (**a**), continuum removal (CR; (**b**)), standard normal variate transformation (SNV; (**c**)), and first derivative transformation (FD; (**d**)).

To mitigate the instability caused by the SNR at the spectral edges, only the reflectance data within the 400–900 nm wavelength range were extracted for subsequent analysis. This spectral interval covers the strong absorption characteristics of chlorophyll as well as the structure-sensitive information in the red edge region, making it a critical spectral range for chlorophyll content inversion. The raw reflectance spectra were smoothed using a Savitzky–Golay (SG) filter to improve spectral signal-to-noise characteristics. Subsequently, three different transformation processes were applied to the spectral data, including Continuum Removal (CR), Standard Normal Variate (SNV) transformation, and First Derivative (FD) transformation, aiming to enhance the chlorophyll-related absorption and reflection features. Figure 8 illustrates comparisons among these transformation methods. These three preprocessing methods can provide complementary representations of canopy spectral responses under shading conditions. Specifically, CR emphasizes absorption-related features, SNV helps reduce scattering and baseline effects, and FD enhances local spectral-shape variation, especially in chlorophyll-sensitive regions such as the green peak and red-edge domains.

Different preprocessing methods generate multiple sets of high-dimensional features. After merging, the data dimensions increase substantially, leading to potential issues of information redundancy and multicollinearity, thereby undermining model stability and generalization. To address this issue, a two-stage feature selection strategy was adopted in this study: (1) Correlation screening: The Pearson correlation coefficient between each spectral variable and the measured chlorophyll was calculated to eliminate a large number of irrelevant or weakly correlated features; (2) Boruta feature selection: Building on the retained variables, the Boruta algorithm was further employed for wrapper-based feature screening [59]. This algorithm is implemented based on the random forest model using the idea of “shadow features”.

### 4.4. RTM-Based Spectral Simulation

The canopy reflectance spectra under various combinations of physiological and structural parameters were simulated based on the PROSAIL radiative transfer model to establish a physically constrained spectral–chlorophyll mapping relationship. The value ranges of each parameter in PROSAIL were set according to the existing literature and the observation data from this experiment [20,60], ensuring that the simulations both span plausible theoretical variability and remain consistent with physiological constraints. A large number of parameter combinations were generated by Latin hypercube sampling of the parameter space, and the corresponding reflectance spectra were forward-simulated using PROSAIL to construct a spectral–chlorophyll lookup table. This simulated data served as the source domain data for subsequent domain-adaptive inversion modeling.

### 4.5. CAI-DAI Framework Architecture

**Figure 9:**
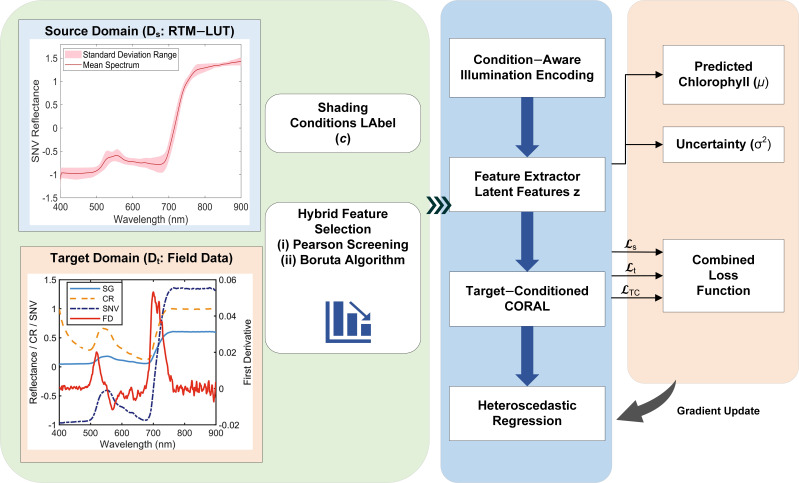
CAI-DAI framework for chlorophyll content retrieval in *H. hainanensis*.

Building on the conditional distribution shift between RTM-simulated spectra and field measurements arising from shading-induced changes in the illumination context, we developed the Condition-Aware Illumination Domain-Adaptive Inversion (CAI-DAI) inversion framework (Figure 9). Here, illumination refers to the shading-modulated light environment associated with different shade conditions. This framework aims to improve the accuracy and cross-condition consistency of chlorophyll inversion under realistic shaded scenarios, while maintaining the physical consistency of the simulated domain. It integrates mechanism that combines condition-aware representation learning, conditional distribution alignment, and uncertainty-robust learning. Specifically, through the illumination-condition encoding module, the four discrete shading levels were mapped into 32-dimensional high-level semantic vectors using an embedding layer. The selected spectral features were then transformed into 256-dimensional deep features by the FeatureExtractor, after which the two representations were fused. Dropout was set to 0.2 in both modules for regularization. The training batch size was 64.

#### 4.5.1. Condition-Aware Illumination Encoding

In CAI-DAI, the shading condition is introduced as an explicit input. An illumination embedding module is introduced to map discrete shading levels *c* into continuous vector representations. These embeddings are subsequently integrated with spectral features within a residual-structured feature extractor to learn latent representations:

(2) $$z=f_{\theta }(x,e(c))$$

The illumination embedding $e\left(c\right)$ is fused with spectral features during feature extraction, enabling the network to dynamically modulate its feature responses according to the illumination context associated with the current shading regime. Conceptually, this mechanism elevates the “shading effect” from an implicit nuisance factor to a learnable conditioning variable, thereby mitigating the disruptive impact of conditional shift on the reflectance–chlorophyll inversion mapping.

#### 4.5.2. Target-Conditioned CORAL

The CAI-DAI framework narrows the distribution discrepancy between the simulated domain ($D_{s}$) and the measured domain ($D_{t}$) by introducing Correlation Alignment (CORAL) in the latent feature space z. Let the feature covariance matrices of the source domain and target domain be $C_{s}$ and $C_{t}$, respectively, with a dimension of d. The standard CORAL loss is then defined as:

(3) $$L_{\mathrm{CORAL}}=\frac{1}{4d^{2}}{∥C_{s}-C_{t}∥}_{F}^{2}$$

The standard CORAL loss is extended in this study to a Target-Conditioned CORAL designed for heterogeneous illumination conditions. This modification avoids the risk of negative transfer caused by “mixed alignment” across different shading levels, enabling the model to learn a representational structure closer to the physical consistency of the source domain under each shading level. The calculation formula is as follows:

(4) $$L_{\mathrm{TC}\text{-}\mathrm{CORAL}}=\frac{1}{|C|}\sum_{c\in C}\frac{1}{4d^{2}}{∥C_{s}-C_{t}^{\left(c\right)}∥}_{F}^{2}$$

#### 4.5.3. Uncertainty Weighting

A heteroscedastic regression head is adopted in CAI-DAI to jointly output the predictive mean μ and the log-variance σ. The loss function in the form of heteroscedastic negative log-likelihood defined as follows:

(5) $$L_{\mathrm{het}}=\frac{1}{N}\sum_{i=1}^{N}\left[exp(-log\sigma_{i}^{2}){\left(y_{i}-\mu_{i}\right)}^{2}+log\sigma_{i}^{2}\right]$$

Furthermore, condition-level uncertainty weighting is introduced to enable robust optimization across shading conditions.

(6) $$L_{t}=\sum_{c\in C}\left[exp(-logv_{c})\cdot E_{i\in c}[l_{i}]+logv_{c}\right]$$

where $E_{i\in I_{c}}[l_{i}]$ denotes the empirical expectation of the per-sample loss under condition c. This mechanism allows the contribution of each condition to the gradient updates to be adaptively reweighted, thereby improving cross-condition consistency.

This uncertainty weighting design helps account for condition-dependent heterogeneity during optimization. Different shading conditions may have different error variance and sample complexity, so treating all samples and conditions as equally reliable can lead to unstable updates or overemphasis on harder subsets. By introducing heteroscedastic prediction and condition-level reweighting, the model can optimize more robustly across heterogeneous shading scenarios.

### 4.6. Data Partitioning and Model Evaluation

The look-up table generated by PROSAIL simulations was used as the source domain data, which were randomly partitioned into training and validation sets at an 8:2 ratio to learn the underlying physical mapping between spectral reflectance and chlorophyll content. The in situ measured spectra were used as the target domain data. In the target domain, the measured samples were split into fine-tuning and test subsets using stratified random sampling by shading level (S1–S4). This ensured that both subsets contained samples from different shading conditions and prevented any single shading level from being missing or strongly imbalanced in either subset. In addition, the two subsets were kept mutually exclusive at the individual-plant level, so that the same plant did not appear in both model adaptation and independent testing. To systematically assess how different data splitting strategies affect transfer performance, multiple fine-tuning–test split ratios were considered, including 3:7, 4:6, 5:5, 6:4, and 7:3. This design enabled evaluation of model behavior under different levels of labeled target-domain data, ranging from limited-label adaptation to relatively better-labeled conditions [23].

To comprehensively evaluate the contribution of each component within the CAI-DAI framework, ablation configurations were constructed. All models were trained using the same preprocessing pipeline and the same feature-selection outputs. The backbone architecture was a deep residual multilayer perceptron (Deep Residual MLP; ResDNN) [61]. The compared variants were defined as follows: (1) ResDNN: Without any domain adaptation mechanism, it serves as a performance benchmark to evaluate the capability of pure data-driven models under cross-domain conditions. (2) Global Domain Alignment (GAI): Global CORAL domain alignment was introduced to assess the effect of “overall statistical distribution alignment” in cross-domain inversion. (3) Condition-Aware Adaptation (CA): Domain alignment with target conditional constraints was adopted to verify the ability of conditional constraint alignment to mitigate conditional shift. (4) CA and Illumination Encoding (CA-IE): Shading level encoding embedding was added on the basis of conditional adaptation to explicitly model the impact of shading levels on the spectrum–chlorophyll relationship. (5) CAI-DAI: The full framework integrates condition-aware representation learning, conditional distribution alignment, and uncertainty-aware robust learning. To comprehensively evaluate the prediction accuracy and stability of the models, three metrics were adopted: coefficient of determination (R^2^), normalized root mean square error (nRMSE), and mean absolute error (MAE). Data preprocessing, statistical analysis, and figure generation were conducted in MATLAB R2023a (MathWorks, Natick, MA, USA). Model development and training were performed in Python 3.12. The computations were run on a workstation equipped with an NVIDIA GeForce RTX 4070 Ti SUPER GPU (16 GB VRAM), an AMD Ryzen 9 7900X3D 12-core processor, and 32 GB RAM.

## 5. Conclusions

This study focused on the robust estimation of chlorophyll content in *H. hainanensis* under shade management. The effects of shading gradients on chlorophyll content and canopy spectra of *H. hainanensis* were systematically revealed. A conditional-aware domain adaptation framework (CAI-DAI) was proposed and validated for cross-shading generalization. The results showed that chlorophyll content in *H. hainanensis* leaves increased with rising shading intensity. Clear differences were observed in canopy spectral distributions among different shading levels. In cross-shading estimation tasks, models integrating conditional information achieved better performance than the comparative methods. The CAI-DAI model maintained the highest and most stable test accuracy across fine-tuning ratios (30–70%). Its MAE ranged from 4.355 to 4.774 μg·cm^−2^. Compared with the baseline ResDNN model, the MAE was reduced by 28.4–49.1%. Moreover, CA-IE and CAI-DAI yielded more stable predictions under all fine-tuning ratios than ResDNN, GAI, and CA models. Further evaluation at individual shading levels (S1–S4) showed that the MAE ranges of CAI-DAI were lower than those of CA-IE. CAI-DAI also showed smaller coefficients of variation for MAE under most fine-tuning ratios. This reflects that conditional and uncertainty modeling can help improve consistency in cross-shading prediction.

Overall, this study suggests that explicit conditional modeling and conditional alignment can alleviate shading-induced cross-condition differences and improve estimation robustness within the scope of the present dataset and controlled experimental setting. The CAI-DAI framework maintained stable performance even under limited sample conditions. It provides a promising methodological framework for chlorophyll monitoring and management in controlled shade-management scenarios. However, the current conclusions should be interpreted with caution, as the study was conducted on a single shade-tolerant tree species under controlled shading conditions. Future research may extend discrete shading levels to continuous light-environment representations. Extrapolation ability can be validated across more species and growth stages. Combination with radiative transfer constraints can also be explored to improve interpretability across scenes.

## Figures and Tables

**Table 1:** PERMANOVA results for spectral differences in CR, SNV, and FD transformations among shading levels.

Spectral Transformations	Region	Pseudo-F	*p*_perm_	R^2^_perm_
CR	Green peak	8.689	0.001	0.077
CR	Red edge	24.264	0.001	0.190
SNV	Green peak	46.885	0.001	0.311
SNV	Red edge	68.639	0.001	0.398
FD	Green peak	44.968	0.001	0.303
FD	Red edge	76.013	0.001	0.423

**Table 2:** Comparison of test accuracies (nRMSE and MAE) among five models (ResDNN, GAI, CA, CA-IE, CAI-DAI) under different fine-tuning set ratios (30–70%).

Model/Ratio	Metrics	3:7	4:6	5:5	6:4	7:3
ResDNN	nRMSE (%)	32.1	32.4	26.6	25.1	22.9
	MAE (μg·cm^−2^)	8.706	8.79	7.222	6.599	5.951
GAI	nRMSE (%)	34.3	31.6	26.5	23.1	21.8
	MAE (μg·cm^−2^)	9.442	8.475	7.126	5.905	5.512
CA	nRMSE (%)	30.8	28.6	26.2	23.6	21.6
	MAE (μg·cm^−2^)	8.485	7.747	7.004	6.338	5.607
CA-IE	nRMSE (%)	18.7	17.3	18.3	17.4	17.3
	MAE (μg·cm^−2^)	5.024	4.667	4.934	4.607	4.600
CAI-DAI	nRMSE (%)	18.2	16.5	17.9	16.4	16.4
	MAE (μg·cm^−2^)	4.761	4.516	4.774	4.355	4.502

**Table 3:** MAE values of chlorophyll content estimation for *H. hainanensis* under different shading levels (S1–S4) by the CA-IE and CAI-DAI models at different fine-tuning set ratios.

Model	Ratio	S1	S2	S3	S4
CA-IE	3:7	4.463	5.643	4.853	5.803
	4:6	4.405	4.918	4.837	4.828
	5:5	4.581	5.319	4.989	5.273
	6:4	4.355	3.979	5.578	4.782
	7:3	4.072	6.058	4.622	4.396
CAI-DAI	3:7	4.454	5.182	4.462	5.307
	4:6	4.284	4.089	4.753	5.145
	5:5	4.704	4.701	4.931	4.835
	6:4	4.476	3.504	4.330	4.887
	7:3	4.129	4.721	4.275	5.267

## Data Availability

The data presented in this study are available on request from the corresponding author. The data are not publicly available due to confidentiality requirements associated with multiple collaborating parties involved in the funded project..

## Supplementary Materials

The following supporting information can be downloaded at https://www.mdpi.com/article/10.3390/plants15081236/s1, Figure S1: Feature selection results of the two-stage method.
