# Supplementary figures and images for: A Condition-Aware Shading Domain-Adaptive Framework for Robust Chlorophyll Inversion Across Shade Managements in Hopea hainanensis
Source: Plants (Basel). 2026 Apr 17;15(8):1236. doi: 10.3390/plants15081236 (PMC13119434; doi:10.3390/plants15081236)

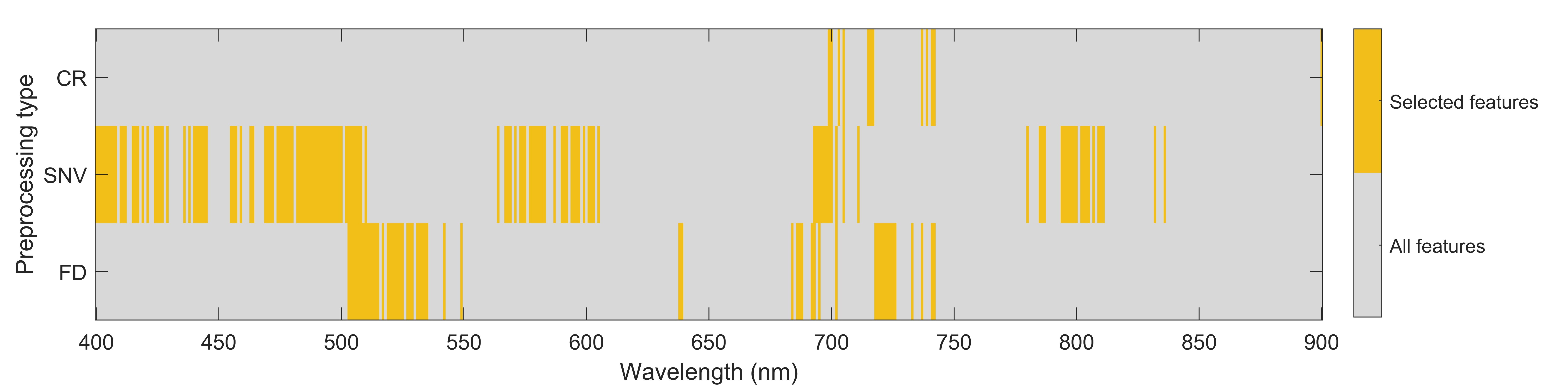

Supplement: Supplementary file 1 [file plants-15-01236-s001.zip › plants-4201622-supplementary.jpg]
